# Supplementary material for: Defining expansions and perturbations to the RNA polymerase III transcriptome and epitranscriptome by modified direct RNA nanopore sequencing
Source: Nat Commun. 2026 Jan 6;17:143. doi: 10.1038/s41467-025-68230-1 (PMC12775064; doi:10.1038/s41467-025-68230-1)
Supplement: Supplementary file 1 — Supplementary Information [file 41467_2025_68230_MOESM1_ESM.pdf]

Defining expansions and perturbations to the RNA polymerase III transcriptome and epitranscriptome by modified direct RNA nanopore sequencing

Supplementary information

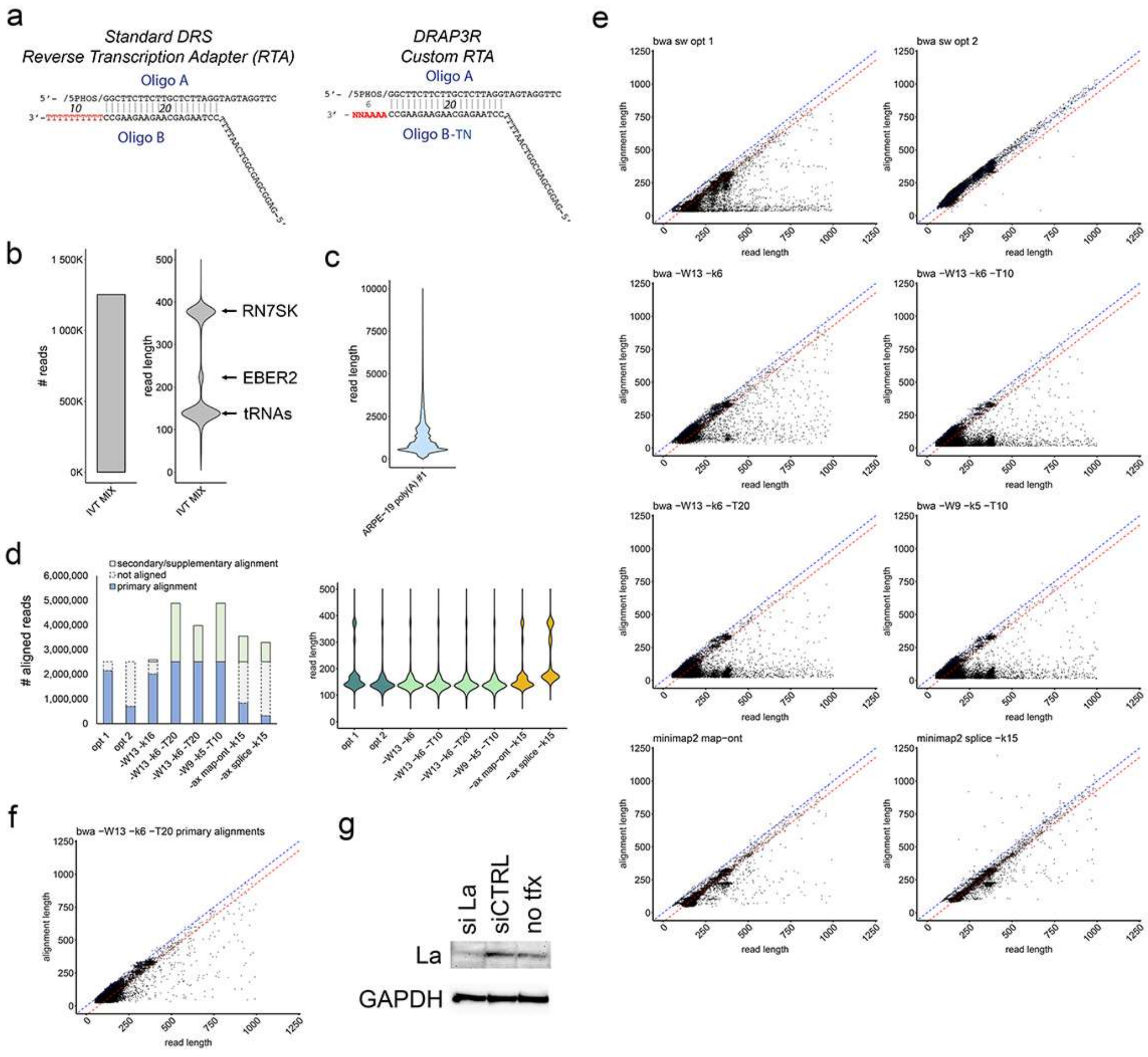

Supplementary Figure S1: DRAP3R efficiently captures poly(U) RNAs.

(a) The standard double-stranded DNA nanopore poly(T) adaptor (left) is shown in contrast to our custom poly(U) targeting DRAP3R adaptor (right). **(b)** Total read count and read length distribution for a DRAP3R dataset derived from a pool of in vitro transcribed (IVT) RNAs comprising RN7SK and EBER2 RNAs along with the pre-tRNAs tRNA-Arg-ACG-1-1, tRNA-

Glu-TTC-2-1, and tRNA-Glu-CTC-1-1 using RA flowcells with RNA004 chemistry and a minimum read length cutoff of 20 nt. **(c)** read length distribution for the standard DRS run on ARPE-19 poly(A) RNA. **(d)** Sequence reads derived from the ARPE-19 #1 DRAP3R sample were aligned to the HG38 genome using BWA or minimap2 in combination with different flags. Total numbers of primary, secondary/supplementary, and unaligned reads are shown on the left with the read length distribution of aligned reads shown on the right. **(e)** Scatter plots comparing read length vs. alignment length for each alignment strategy used in (d). Optimal results should show the aligned length of reads is generally 60-70nt shorter than the full read length, indicative of full alignment along the full RNA body and excluding the untrimmed adapter sequence. **(f)** Filtering to retain only primary alignments resulting from the optimal alignment strategy (bwa mem –W 13 –k 6 –T 20 –x ont2d) further increased the proportion of read alignments that produce full length alignments, minus the adapter sequence. **(g)** Western blot showing La protein and GAPDH levels in ARPE-19 cells treated with a siRNA targeting La protein, as well as mock transfected and untransfected controls.

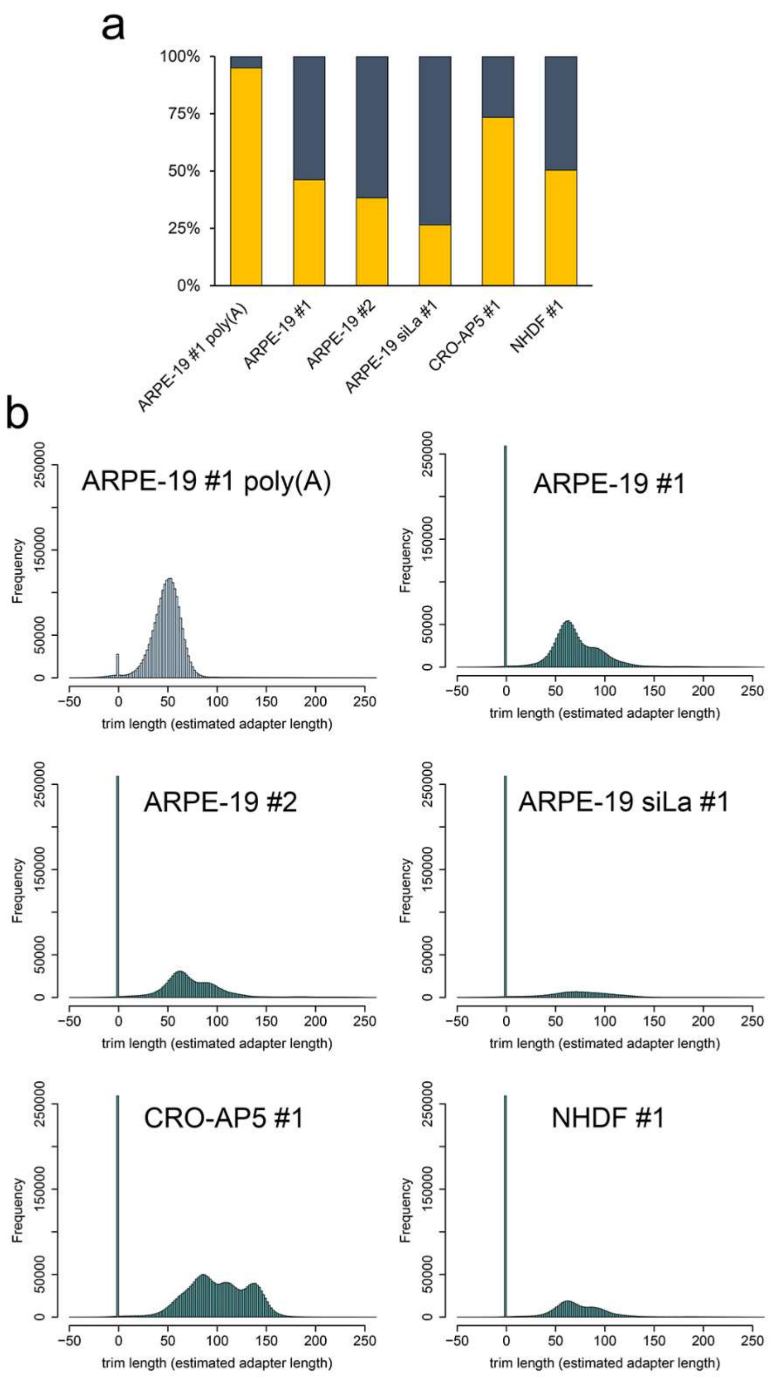

Supplementary Figure S2: DRAP3R efficiently captures poly(U) RNAs.

(a) Barplot denoting the proportion (blue/green) of reads that could be trimmed for each dataset using dorado v0.7.0. Untrimmed reads (grey) were classified as those in which the difference between trimmed and untrimmed read was < 10 nt. **(f)** Trim length distributions for each dataset were derived by calculating the difference in length for each trimmed read relative to its untrimmed state. A normal distribution with a modal value around 65 nt (adapter length) is observed for the ARPE-19 #1 poly(A) DRS dataset while DRAP3R datasets show multi-modal distributions resulting from over-trimming of reads.

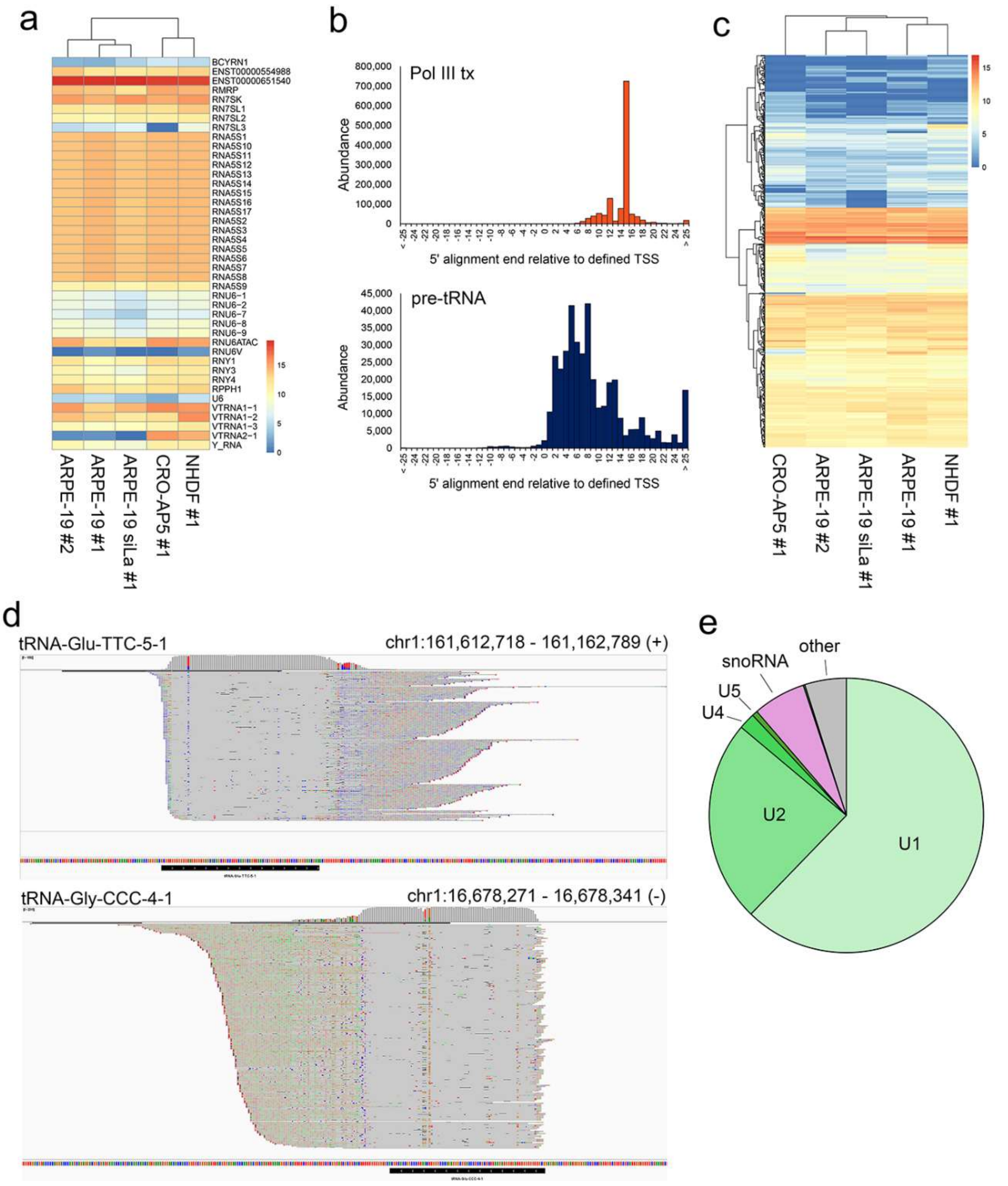

Supplementary Figure S3: Further analysis of poly(U) RNAs captured by DRAP3R.

(a) heatmap denoting, for each dataset, the abundances (log10 TPM) of RNAs derived from Pol III genes. **(b)** barplots denoting the 5' alignment positions against (top) Pol III genes and (bottom) Pol III tRNA genes. **(c)** heatmap denoting, for each dataset, the abundances (log10 TPM) of RNAs derived from Pol III tRNA genes. **(d)** Integrative Genomics Viewer (IGV) screenshots showing extensive primary alignments across two low-confidence tRNA genes; tRNA-Glu-TTC-5-1 and tRNA-Gly-CCC-4-1. Soft-clipped (adapter) sequences are shown to the right (tRNA-Glu-TTC-5-1) or left (tRNA-Gly-CCC-4-1) of the alignment. **(e)** Breakdown of Pol II transcribed poly(U) RNAs detected by DRAP3R for sample ARPE-19 #1.

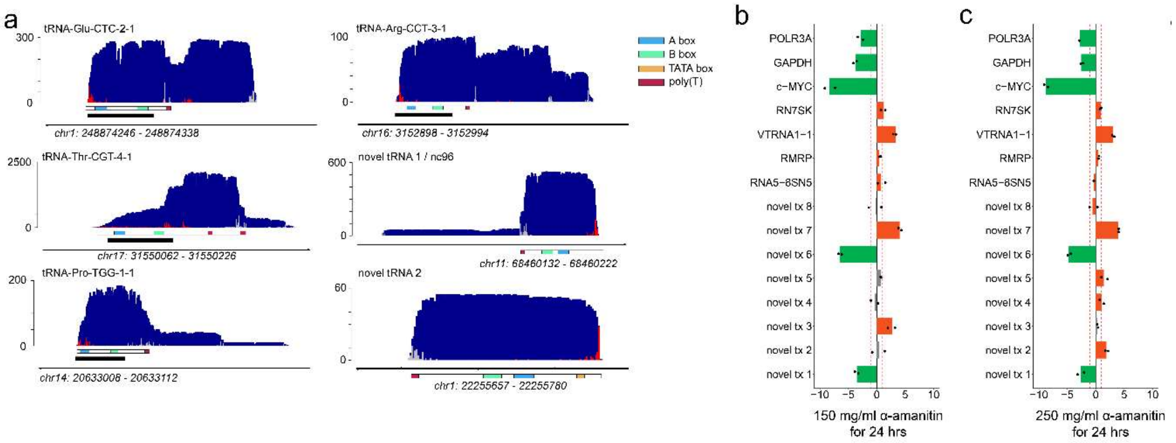

Supplementary Figure S4: DRAP3R expands the RNA polymerase III transcriptome

(a) Coverage plots for four recently confirmed and two recently predicted tRNA genes are shown in dark blue with 5’ (red) and 3’ (grey) alignment ends (indicating putative sites of transcription initiation and 3’ end processing) shown as overlaid histograms. Characteristic sequence features including A (blue) and B (green) boxes as well as TATA boxes (pink) and poly(T) tracts ≥ 4 nt (purple) are shown as part of the underlying transcript schematic. Genome co-ordinates are specified for the HG38 assembly while the y-axis denotes read depth. **(b-c)** ARPE-19 cultures were treated with 150, or 250 µg/ml α-amanitin to selectively inhibit Pol II transcription and quantitative RT-PCR performed on extracted RNA to determine expression levels relative to 18s rRNA (expressed as log2 fold difference).

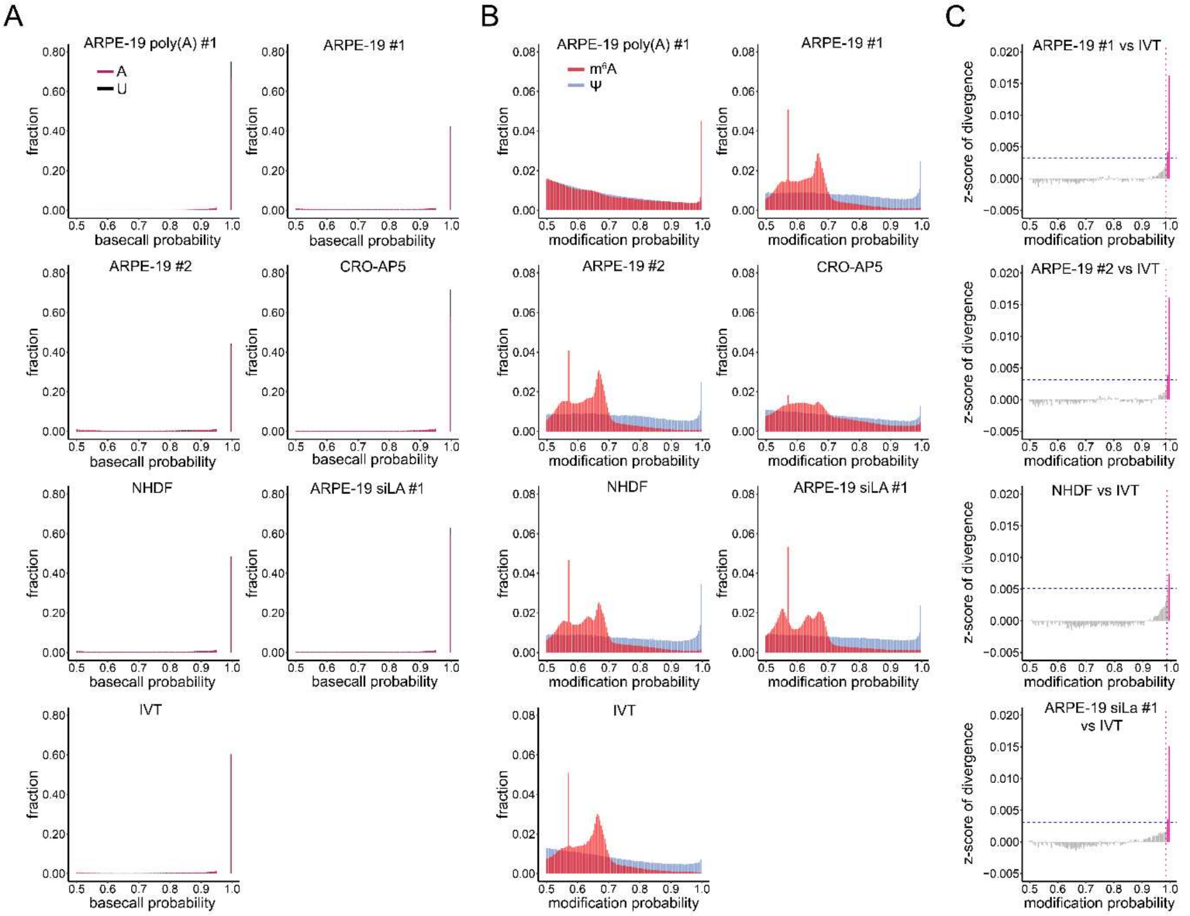

Supplementary Figure S5: Modification probability distributions for all datasets

Histograms denoting basecall probability distributions for **(a)** U (blue) and A (red) and **(b)** Ψ (blue) and m^6A (red) for each individual dataset shown in Fig. 1 and the pooled IVT dataset shown in Supplementary Fig. 1b. **(c)** Visualization of z-scores derived from a quantitative divergence analysis that compares pseudouridine basecall probability distributions across binned data. significant bins (|z| > 2 relative to the mean difference) are highlighted in pink. Dashed horizontal lines denote the ±2 z-score cutoff, and the dotted vertical line marks the modification probability threshold (0.98).

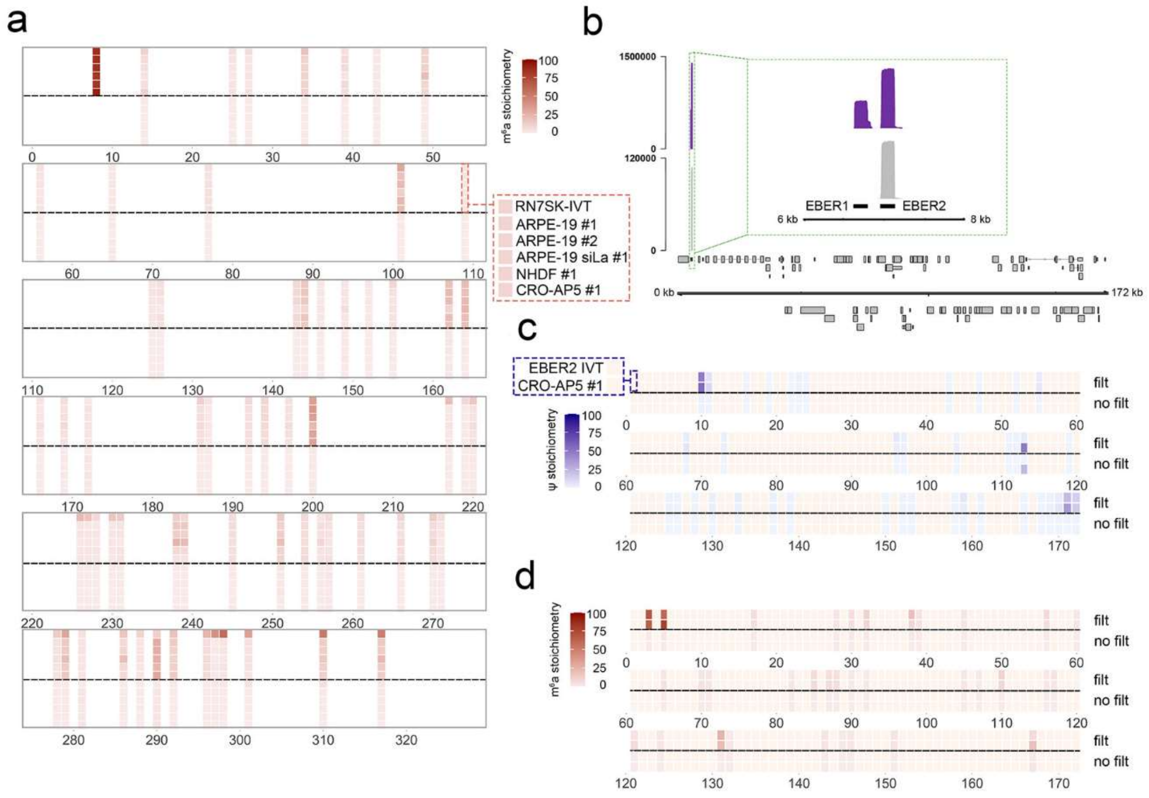

Supplementary Figure S6: Detection of m^{6}A and Ψ in pre-mature Pol III transcribed RNAs

(a) m^{6}A stoichiometry plot for pre-mature RN7SK demonstrates a high-false positive rate in unfiltered datasets (top five rows, unmodified sites shown in pale blue) whereas filtered datasets (bottom five rows) show absence of co-transcriptional m^{6}A installation. Increasing stoichiometry is shown by increased blue (Ψ) or red (m^{6}A) shading. **(b)** Coverage plots of EBER2 in the DRAP3R CRO-AP5 and EBER2 IVT datasets. Y-axis denotes read depth while X-axis denotes genome co-ordinates. The EBER2 gene annotation is shown as a black box. **(c)** Ψ and **(d)** m^{6}A stoichiometry plots for pre-mature EBER2 also demonstrate a high-false positive rate in unfiltered datasets (upper two rows, unmodified sites shown in pale blue) whereas filtered datasets (bottom two rows) show absence of co-transcriptional Ψ and m^{6}A installation.

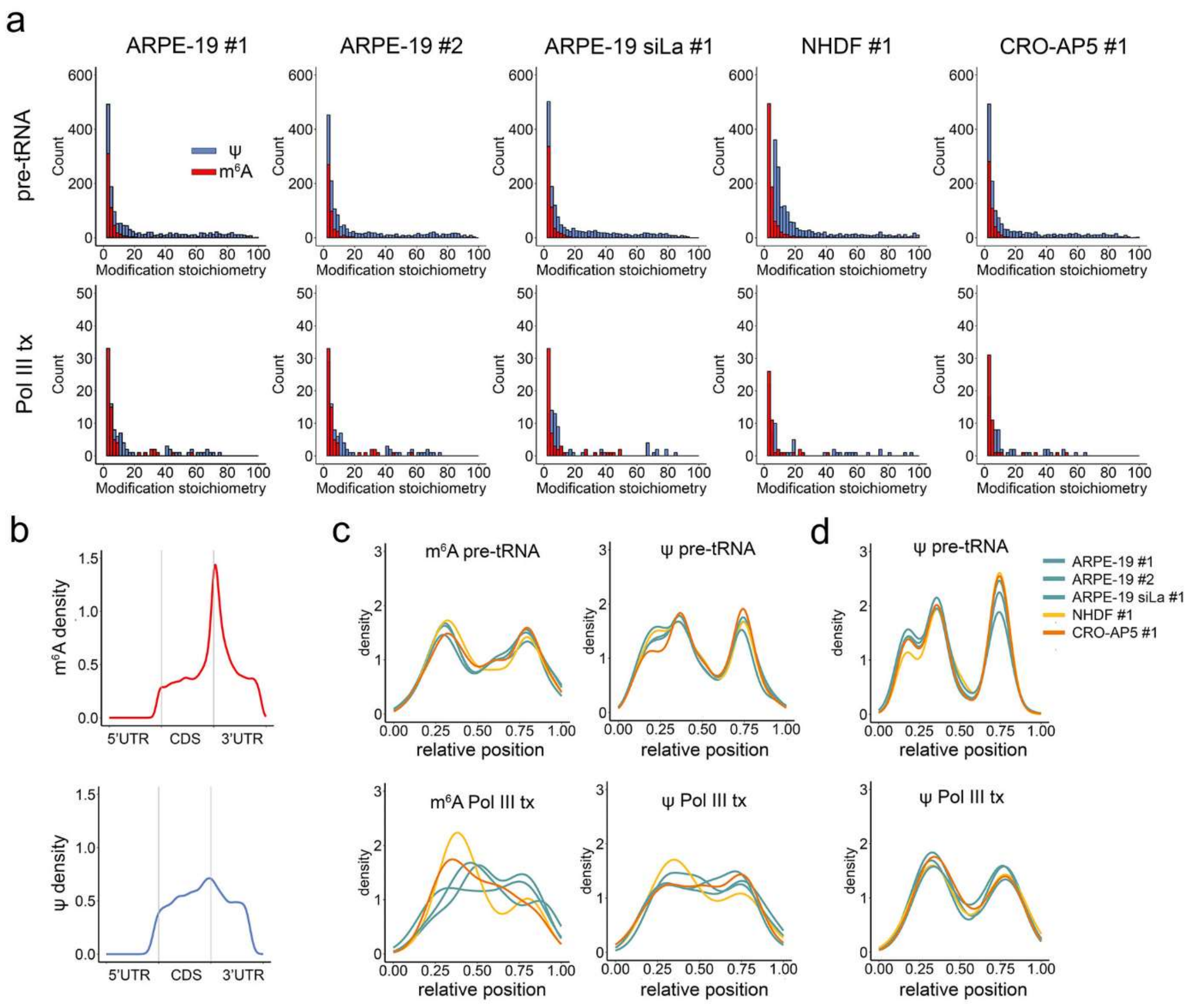

Supplementary Figure S7: Distributions of m^{6}A and Ψ in pre-mature Pol III transcribed RNAs

(a) Stoichiometry distribution histograms for Ψ (blue) and m^{6}A (red) for all filtered DRAP3R datasets. Pol III transcribed pre-tRNAs are shown separately from all other Pol III transcribed RNAs. **(b)** Modification density plots for m^{6}A (top) and Ψ (bottom) recapitulate known distributions of these modifications on polyadenylated RNAs. **(c)** Modification density plots (metaplots) for Ψ and m^{6}A sites at all stoichiometries show consistent and specific distributions for pre-tRNAs and other Pol III transcribed RNAs. **(d)** Modification density plots (metaplots) for Ψ sites with ≥ 10% stoichiometry on pre-tRNAs (top) and other Pol III transcribed RNAs (bottom).

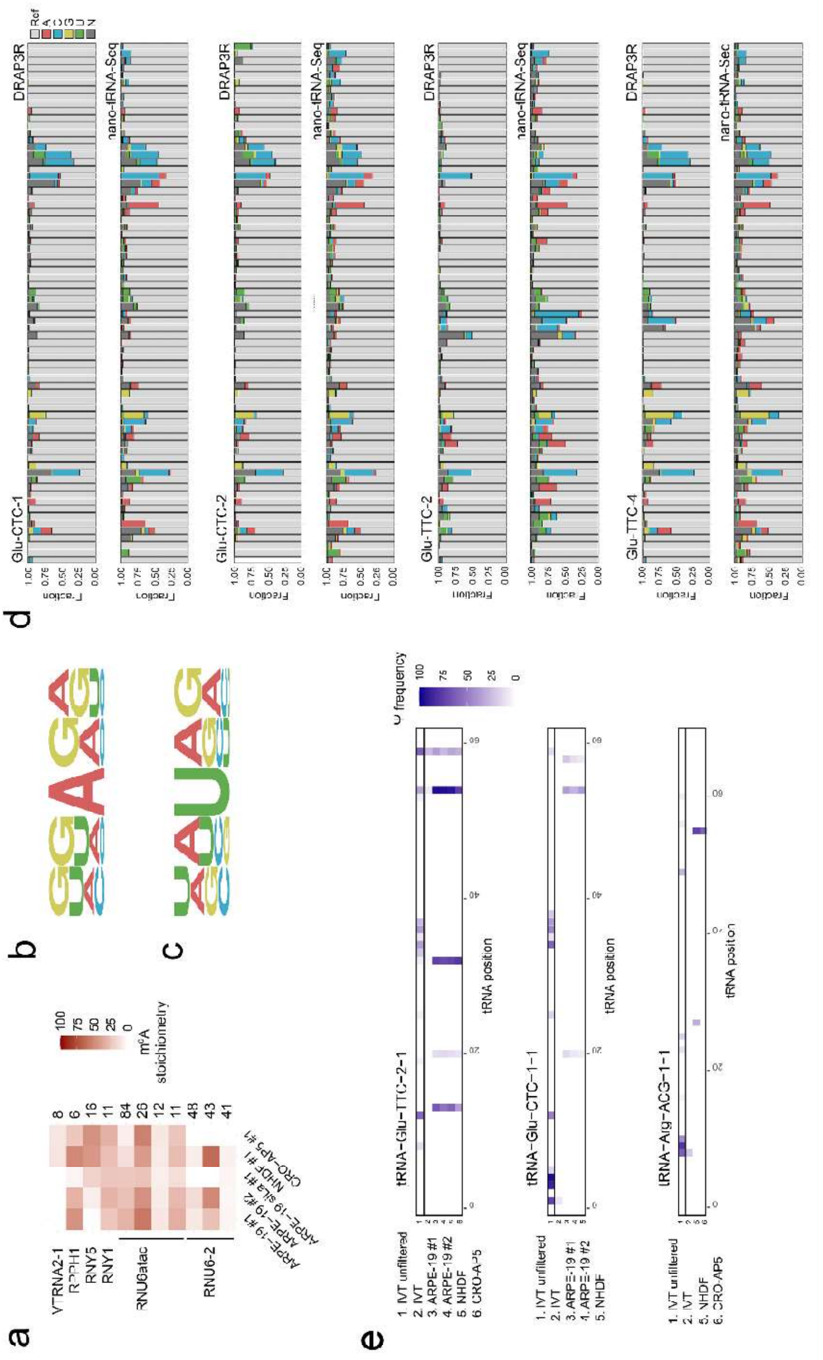

Supplementary Figure S8: Ψ and m^{6}A profiles across the Pol III transcriptome

(a) Heatmap showing stoichiometry distributions across m^{6}A sites in pre-mature Pol III transcribed ncRNAs vary between cell types. **(b-c)** Motif analysis reveals no consensus sequences motifs for m^{6}A **(b)** and Ψ **(c)** installation on Pol III transcribed ncRNAs (excluding pre-tRNAs). **(d)** Distribution of basecall errors for the ARPE-19 #1 DRAP3R and nano-tRNA-Seq datasets across four glutamic acid isodecoders. **(e)** Ψ profiles across three pre-tRNAs with coverage ≥ 50 reads for filtered DRAP3R datasets ARPE-19 #2, ARPE-19 + siLa #1, NHDF #1, and CRO-AP5 #1 as well as filtered and unfiltered versions of the pooled IVT dataset.

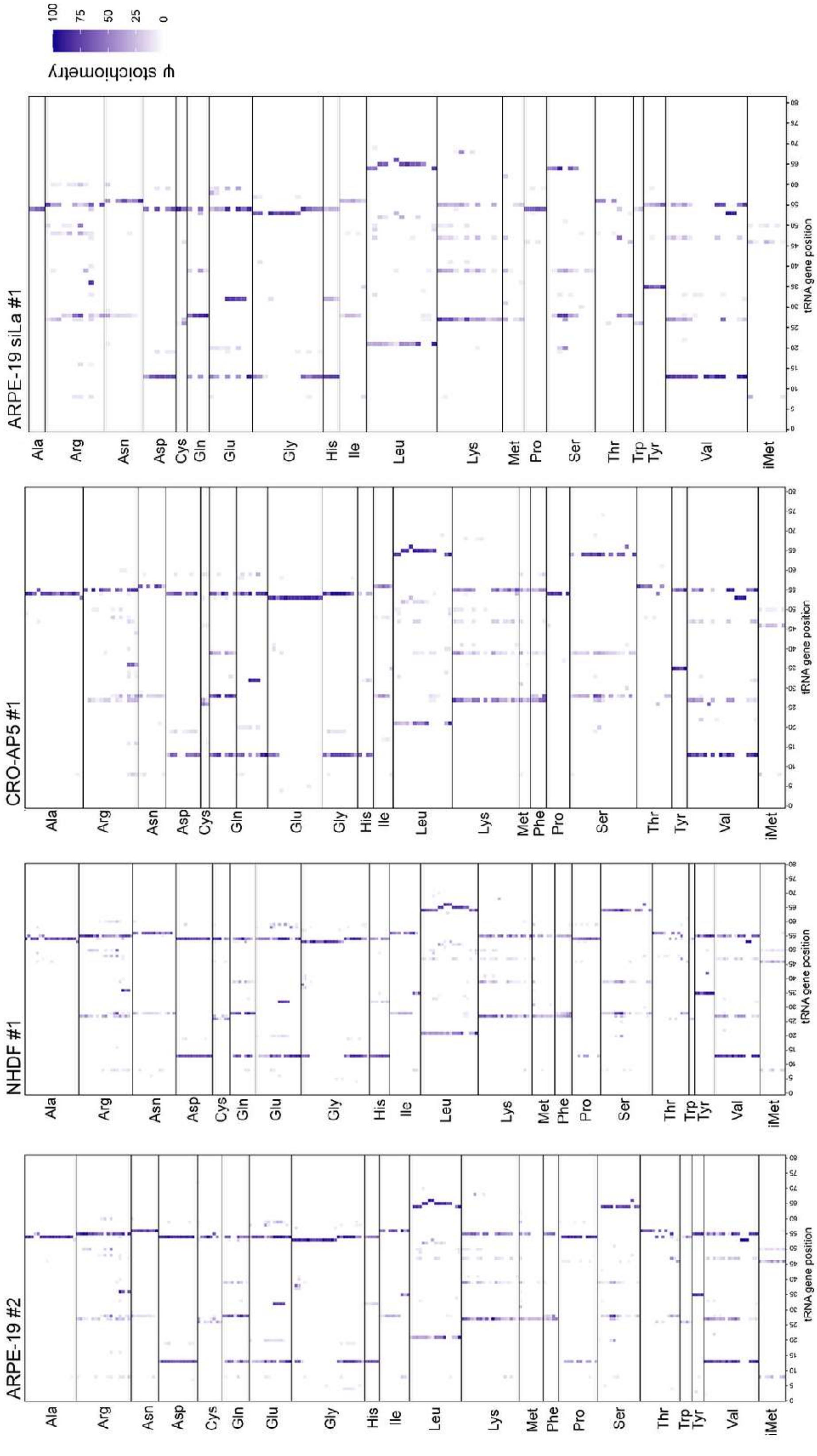

Supplementary Figure S9: Ψ profiles across all pre-mature **pre-tRNAs**

Ψ profiles across all pre-mature pre-tRNAs with coverage ≥ 50 reads for DRAP3R datasets ARPE-19 #2, ARPE-19 + siLa #1, NHDF #1, and CRO-AP5 #1. Each row depicts different tRNA genes and rows are grouped by isoacceptor.

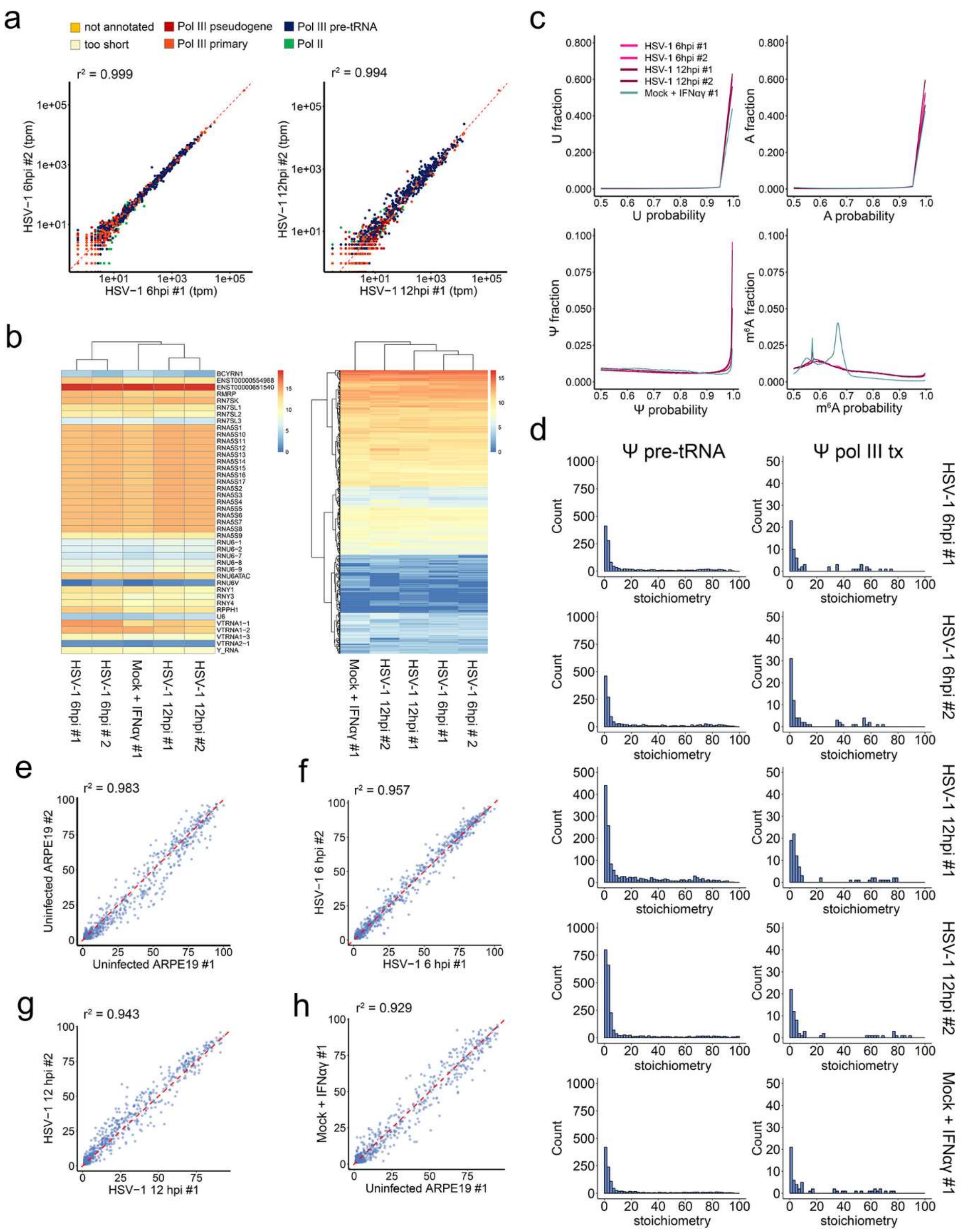

Supplementary Figure S10: Viral infection induced regulation the Pol III transcriptome and Ψ installation

(a) Scatter plots comparing the abundances of RNAs within distinct classes of RNA Pol II and RNA Pol III transcripts shows very high correlation between biological replicates of HSV-1 infected ARPE-19 cells harvested at (left) 6hpi and (right) 12hpi. **(b)** heatmaps denoting, for each dataset, the abundances (log10 TPM) of RNAs derived from (left) Pol III genes and (right)

Pol III tRNA genes. **(c)** Individual basecall probability distributions for U, A, Ψ and m^6A for each infected cell dataset. **(d)** Stoichiometry distribution histograms for Ψ in the filtered HSV-1 infected ARPE-19 datasets. Pol III transcribed pre-tRNAs are shown separately from other Pol III transcribed RNAs. **(e-h)** Scatter plot comparing Ψ stoichiometries on pre-tRNAs show very high correlation between biological replicates of **(e)** uninfected ARPE-19 cells, **(f)** HSV-1 infected ARPE-19 cells harvested at 6hpi, **(g)** HSV-1 infected ARPE-19 cells harvested at 12hpi, and **(h)** uninfected ARPE-19 cells versus uninfected ARPE-19 cells treated with a combination of interferons α and γ.

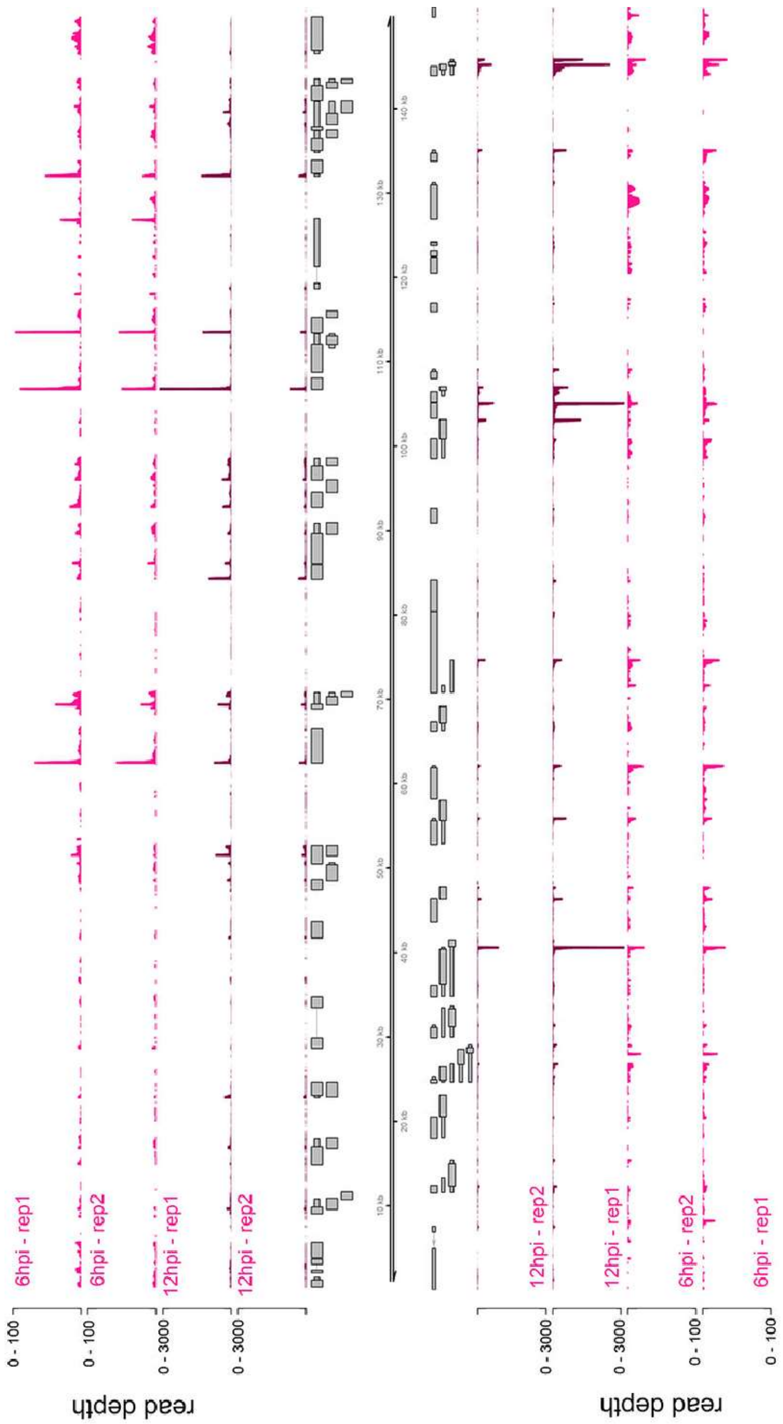

Supplementary Figure S11: Putative Pol III transcription from the HSV-1 genome

Strand-specific coverage plots across the HSV-1 strain KOS genome for all biological replicates of HSV-1 infected ARPE-19 cells harvested at 6 (deep pink) and 12 hpi (purple). The Y-axis denotes depth of coverage. HSV-1 gene structures are shown in grey with canonical CDS regions indicated by wide boxes and UTRs shown as thin boxes.

Dataset	RNA Pol III				no annotation	too short	Viral	Pol II	Total
	SNARs	Pol III pre-tRNA	Pol III primary	Pol III pseudogene					
ARPE-19 poly(A) #1	65	1,919	5,768	15,906	23,532	161,881		1,693,424	1,902,495
ARPE-19 #1	130	637,510	1,599,122	19,830	111,222	72,973	0	73,493	2,514,280
ARPE-19 #2	342	548,919	1,240,504	20,472	108,326	65,895	0	37,310	2,021,768
ARPE-19 siLa #1	28	146,265	594,172	6,291	103,057	29,168	0	26,578	905,559
ARPE-19 + IFNag #1	876	313,824	854,489	10,251	74,843	41,211	0	14,536	1,310,030
NHDF #1	7,821	383,851	413,576	9,266	51,607	29,249		24,099	919,469
CRO-AP5 #1	61,602	182,332	188,618	4,511	62,670	39,364	2,046,140	8,723	2,593,960
HSV-1 6 hpi #1	509	635,543	1,068,605	14,571	80,277	10,097	1,855	26,494	1,837,951
HSV-1 6 hpi #2	444	729,443	1,229,756	18,413	100,841	21,346	1,088	29,411	2,130,742
HSV-1 12 hpi #1	2,679	1,330,464	1,994,444	41,096	112,318	25,766	41,718	29,643	3,578,128
HSV-1 12 hpi #2	463	302,582	412,798	5,190	91,658	39,999	8,413	4,680	865,783
HSV-1 12 hpi #3	1,222	401,691	625,482	11,795	111,534	46,761	13,925	9,117	1,221,527

Supplementary Table S1: Read classifications and abundances

transcript	ensembl_gene	symbol
ENST00000303910.5	ENSG00000277075	H2AC8
ENST00000331380.4	ENSG00000184260	H2AC20
ENST00000331491.2	ENSG00000183598	H3C13
ENST00000341023.2	ENSG00000196866	H2AC7
ENST00000343677.4	ENSG00000187837	H1-2
ENST00000356950.2	ENSG00000197903	H2BC12
ENST00000361390.2	ENSG00000198888	ND1
ENST00000361739.1	ENSG00000198712	COX2
ENST00000362079.2	ENSG00000198938	COX3
ENST00000369159.3	ENSG00000288825	H2AC18
ENST00000372491.4	ENSG00000188483	IER5L
ENST00000377727.2	ENSG00000158406	H4C8
ENST00000377803.4	ENSG00000197061	H4C3
ENST00000391418.3	ENSG00000212724	KRTAP2-3
ENST00000391418.3	ENSG00000292034	
ENST00000391418.3	ENSG00000262862	
ENST00000396984.2	ENSG00000180596	H2BC4
ENST00000438576.3	ENSG00000175573	C11orf68
ENST00000449692.3		
ENST00000457237.1	ENSG00000235444	PSMB3P2
ENST00000508390.1	ENSG00000248530	BCL2L12P1
ENST00000530188.1	ENSG00000175573	C11orf68
ENST00000541455.1	ENSG00000181924	COA4
ENST00000579512.3	ENSG00000270276	H4C15
ENST00000607355.3	ENSG00000288859	H2AC19
ENST00000610747.1	ENSG00000277258	PCGF2
	ENSG00000278644	
ENST00000612966.3	ENSG00000287080	H3C3
ENST00000613854.2	ENSG00000275714	H3C1
ENST00000614911.3	ENSG00000274750	H3C6
ENST00000621411.3	ENSG00000286522	H3C2
ENST00000704027.1	ENSG00000087074	PPP1R15A

Supplementary Table 2: Putative polyuridylated mRNAs

transcript	chr	start	end	strand	sequence (5’ -> 3’)
tRNA-Glu-CTC-2-1	1	248,874,246	248,874,338	+	CTTccctggtggtctagtggttaggattcggcgctctcaccgccgcggcccgggttcgattcccggtcaggaaaGTAAGCCGTTTT
tRNA-Thr-CGT-4-1	17	31,550,062	31,550,226	+	CTGGGCTGTCAGGCGCGGTGGCCAAGTGGTAAGGCGTCGGTCTCGTAAACCGAAGATCGCGGGTTCGAACCCCGTCCGTGCCTGAGACCCGAGGTAGGGCTTTGGCTGTGGGGAAGTCGGGTTTTCTCCACGTACGCCGTCCCTTCTACGTGGCATTTT
tRNA-Pro-TGG-1-1	14	20,633,008	20,633,112	+	CTCGTTGGTCTAGTGGTATGATTCTCGCTTTGGGTGCGAGAGGTCCGGTTCAATCCCGGACGAGCCCTTACTTTCCTTTCCGTTTCATCTTTCTCTCTTT
tRNA-Arg-CCT-3-1	16	3,152,898	3,152,994	+	gccccggtggcctaatggataaggcattggcctcctaagccagggattgtgggttcgagtcccacccgggtaAAGAAGGCCGAATTTT
novel tRNA 1	11	68,460,132	68,460,222	-	CTAGGGAATCAGCTTAAGTGGAGGAGCGTTCGTTTAGTATGTGAGAGGTAACGGGATCGATGCCTGCATTCTCCACAGGGGAATTTT
novel tRNA 2	1	22,255,657	22,255,795	-	ATTTACAGTCCCGGCACTGTGGCTCACGCCTATAATCCCAGCAATTTGGGAGGCCAAGGAGGCAGGATCACTTGGGGCCAAGAGTTCAAGACCAGTTTGGCCAACATAGTAAGACCCTGTCTCTATTTAATACATTTT
novel tx 1 / AluJb SINE Alu	17	17,960,225	17960341	+	ACTAAGCTCTCtggtttagtggttaagagcaccagctctgctgcccacagacctgggttcaatccctgctctgccactgataatccacagaccttgaggaaggtacttgactttt
novel tx 2 / nc111	1	160,925,972	160,926,060	-	gcctgatgctgtggcttagtggataagactctgtcttttcacagtggtggccagggttcaattcccgacttagggaatgagtactttt
novel tx 3	7	18,038,540	18,038,668	+	TAATTGCAGAGGGGcagtttggtgttgtggatatgcacacagactctggtgccagactgcctggtttgaatcctggctcattaacctaagatctgggtgacttggggcaaattacttaacacatttt
novel tx 4	10	49,387,375	49,387,496	+	TGggtcaggcgtggtggctcaggcatgcagtcccaggactttcggatgccaaggtgggcggatcacttgaggttgggagtttgagaccagcctggccaacatgatgaaacccctctctttt
novel tx 5	7	28,405,584	28,405,720	-	TCAAggccaggcgcagtggctcacacctgtaaccacagctctttgggaggctgagacgggaggataacttgagcctgggagttcgagaccagcttgggcaacatagacagcctgagcgtctctacaaaaagttttt
novel tx 6	8	106,864,056	106,864,155	-	ggccgggcgtggtggctcaagcctataatcccagcactttggtaagcagaggtgggtggatgacaaggtcaggagtttgagaccagcagtggccaCTTTT
novel tx 7	16	22,298,375	22,298,625	-	TAACGGcagtatggttaagtgggtaagagcttggacccgagaacaaaactgtgcaggttcaaatcccaccacagctgcttggcatctgtgacccacctgagttgtctgggtttggtattcctccctattttcatccctaaagtagggaaactaagtaccgacctcCAGACCCCTGCGGGGAAGGAGTAAGGACAGGACGCTAATAAACGTAACCTCTGGGAAGGTTTGTTATTACTTGACAACATGTTTT
novel tx 8	12	93,243,600	93,243,720	-	ctgagagccTgtggtctagaggagaaacacggacttgggagtagacagacatgggttctggtaccagttcttccacttccggtgtgcctttgagagagctgcttctttgagctttggtttt

Supplementary Table 3: novel transcripts identified by DRAP3R

ID	Sequence 5’ –> 3’:
qPCR primers	
18srRNA_PF	AGGAATTGACGGAAGGGCACCA
18srRNA_PR	TTATCGGAATTAACCAGACAAATCG
RMRP_F	AAGAAGCGTATCCCGCTGAG
RMRP_R	GCACTGCCTGCGTAACTAGA
RN7SK_qF	CCAGGGTTGATTCGGCTGAT
RN7SK_qR	GATGGTCGTCCTCTTCGACC
Novel tx_1_F	CTCTCTGGTTTAGTGGTTAAGAGCA
Novel tx_1_R	AGGTCTGTGGATTATCAGTGGC
Novel tx_2_F	AAAGTACTCATTCCCTAAGTCGG
Novel tx_2_R	CTGATGCTGTGGCTTAGTGGATA
Novel tx_3-new_F	TGCCAGACTGCCTGGGTTT
Novel tx_3-new_R	TAAGTAATTTGCCCCAAGTCACC
Novel_tx_4_F	ATGCAGTCCCAGGACTTTCG
Novel_tx_4_R	CAGGCTGGTCTCAAACTCCC
Novel_tx_5_F	CAGGCTGTCTATGTTGCCCA
Novel_tx_5_R	GGCTCACACCTGTAACCACA
Novel_tx_6-new-F	GCCACTGCTGGTCTCAAACT
Novel_tx_6-new-R	CGTGGTGGCTCAAGCCTATAA
Novel_tx_7_F	AGCGTCCTGTCCTTACTCCT
Novel_tx_7_R	GACCCACCTGAGTTGTCTGG
Novel tx_8-new_F	AAGCAGCTCTCTCAAAGGCA
Novel tx_8-new_R	ACGGACTTGGGAGTAGACAGA
POLR3A_F	ATGGTGAAGGAGCAGTTCCG
POLR3A_R	CATCCTATGGTCGAGCACCC
c-MYC-F	TACAACACCCGAGCAAGGAC
c-MYC-R	CTAACGTTGAGGGGCATCGT
RNA5-8SN5-F	CTTAGCGGTGGATCACTCGG
RNA5-8SN5-R	AGTGCGTTCGAAGTGTCGAT
VTRNA1-1-F	TGGCTTTAGCTCAGCGGTTA
VTRNA1-1-R	GGGTCTCGAACAACCCAGAC
RNU2-1-F	ATCGCTTCTCGGCCTTTTGG
RNU2-1-R	CCTATTCCATCTCCCTGCTCC
IVT template generation primers	
EBER2-ivt_F	TATTAGTACTTAATACGACTCACTATA AGGACAGCCGTTGCCCTA
EBER2-ivt_R	AAAATAGCGGACAAGCCGAA
RN7SK-ivt-F	TATTAGTACTTAATACGACTCACTATA GGATGTGAGGGCGATCTGG
RN7SK-ivt-R	TTGGATGTGTCTGGAGTCTTGG
tRNA-Arg-ACG-IVT-F	TATTAGTACTTAATACGACTCACTATAGGGGCCAGTGGCGCAATGG
tRNA-Arg-ACG-IVT-R	AAAGTCGAACGAGCCAGCCAGG
tRNA-Glu-TTC-IVT-F	TATTAGTACTTAATACGACTCACTATAGCCGTCTCCCACATGGTCT
tRNA-Glu-TTC-IVT-R	AAAATTTCGGCGTTCCCACACC

tRNA-Glu-CTC-IVT-F	TATTAGTACTTAATACGACTCACTATAGCAGCTTCCCTGGTGGTCT
tRNA-Glu-CTC-IVT-R	AAAAGAAGATGTTACTCTTTCC
**DRAP3R Reverse Transcription Adaptor**	
Oligo A	/5PHOS/GGCTTCTTCTTGCTCTTAGGTAGTAGGTTC
Oligo B-TN	GAGGCGAGCGGTCAATTTTCCTAAGAGCAAGAAGAAGCCAAAANN

Supplementary Table 4: List of primers and oligonucleotides used in this study
