# Supplementary material for: Defining expansions and perturbations to the RNA polymerase III transcriptome and epitranscriptome by modified direct RNA nanopore sequencing
Source: Nat Commun. 2026 Jan 6;17:143. doi: 10.1038/s41467-025-68230-1 (PMC12775064; doi:10.1038/s41467-025-68230-1)
Supplement: Supplementary file 2 — Description of Additional Supplementary Files [file 41467_2025_68230_MOESM2_ESM.pdf]

Description of Additional Supplementary Information

Supplementary Data 1: Decay rate calculations for Pol III transcripts in ARPE-19 cells transfected with nonsilencing or La protein silencing (siLa) RNAs

Supplementary Data 2: DeSeq2 differential transcript expression analysis between mock-infected and HSV-1 infected (6 hpi) ARPE-19 cells.

Supplementary Data 3: DeSeq2 differential transcript expression analysis between mock-infected and HSV-1 infected (12 hpi) ARPE-19 cells.
